# Comparison of preoperative Nutritional Risk Index and Body Mass Index for predicting immediate postoperative outcomes following major gastrointestinal surgery: Cohort-study

**DOI:** 10.1016/j.amsu.2019.10.011

**Published:** 2019-10-15

**Authors:** Nabin Pokharel, Gaurav Katwal, Subodh Kumar Adhikari

**Affiliations:** National Academy of Medical Science (NAMS), Department of Surgical Gastroenterology, Bir Hospital, Mahabaudha, Kathmandu, 44600, Nepal

**Keywords:** Nutritional risk index, Body mass index, Postoperative complications, Length of hospital stay and cost

## Abstract

**Background:**

Malnutrition is a major risk factor for morbidity and mortality following gastrointestinal (GI) surgery. Nutritional Risk Index (NRI) and Body Mass Index (BMI) are the two well-validated tools that are readily available and do not add financial burden to the patients. The study aimed to analyze NRI and BMI as a preoperative nutritional indicator of postoperative complications following GI surgeries.

**Methods:**

It is an observational study, where preoperative nutritional status and early postoperative complications <30 days (infectious or noninfectious) were studied. The patients admitted between July 2015 to May 2017, who underwent major GI surgeries were included in the study. The correlation between NRI and BMI of these patients were evaluated.

**Results:**

The rate of wound infection was 4 (30.7%) out of 13 in severe malnutrition subgroup defined by NRI <83.5 which was found to be statistically significant (*p* = 0.003). However, it was not significant in a subgroup of patients with undernutrition defined by BMI <18.49%. In a subgroup analysis, abnormal NRI was found to be statistically significant (*p* = 0.004) in patients with malignant disease and malnutrition 64 (47.76%) out of 97 (72.3%). The mean NRI (94.49 ± 9.164) better correlated with advancing age (*p* < 0.05) and the correlation coefficient of 0.3100 showed a significant negative correlation. With 10 fold increase in age (r^2^ = 0.096) the likelihood of malnutrition was 9.6% and subsequently increased postoperative complications.

**Conclusion:**

In cases of malignancy and advanced age, NRI is a better predictor of immediate postoperative outcome than BMI.

## Highlights

• Nutritional Risk Index (NRI) and Body mass Index (BMI) were used to screen the preoperative patients.
• NRI and BMI are the two well validated tools which is readily available and does not add financial burden to the patients.
• NRI better correlated with postoperative wound infections and the length of hospital stay.
• The patients with advanced age and malignant diseases were at higher risk of malnutrition and postoperative complications.

## Abbreviations

AUC: Area under the curve
BMI: Body mass index
CDC: Clavién-Dindo Classification
GI: Gastrointestinal
GLIM: Global Leadership Initiative on Malnutrition
ISGPS: International Study Group of Pancreatic Surgery
LOS: length of hospital stay
NAMS: National Institute of Medical Sciences
NRI: Nutritional Risk Index
POD: Postoperative day
POPF: Postoperative pancreatic fistula
RCT: Randomized controlled trial
SPSS: Statistical Package for the Social Sciences
TPN: Total parental nutrition
WHO: World health organization

## 1 Introduction

Malnutrition is a broad term that is used to describe any subacute or chronic imbalance in nutrition that leads to an inflammatory activity, change in a composition, and decrease in function of a body [1]. WHO addresses 3 aspects of malnutrition viz. 1) undernutrition, that includes wasting, stunting and underweight; 2) micronutrient-related malnutrition, that includes deficiency or excess of micronutrients like important vitamins and minerals; and 3) overweight and noncommunicable diseases related to diet and lifestyle such as heart disease, stroke, diabetes, and some cancers [2].

Malnutrition is one of the major risk factors for poor surgical outcomes in patients undergoing abdominal surgery. However, its prevalence and severity are often underestimated. In one of the Canadian study, over 40% of the hospitalized patients were found to be malnourished [3]. They found 1 in 3 was moderately malnourished and 1 in 10 was severely malnourished which increased their length of hospital stay (LOS) and cost. Similar results of the high prevalence of malnutrition 48.1% were found in the Brazilian National Survey. And they concluded that nutritional evaluation tends to be overlooked by the physicians, though the presence of malnutrition affected the postoperative outcomes of these patients [4]. A successful outcome after surgery is highly dependent on the incidence and severity of postoperative complications as well as their prevention and management. In the year 2012, the total global volume of operations was 312.9 million, which is 38.2% more than an estimated 226.4 million operations in 2004 [5]. And this number will increase due to an expanding aging population and improved access to healthcare. In one of the studies from the USA, the authors found that every person will undergo nearly six surgical procedures in their lifetime. Statistically, we are all preoperative [6].

Buzby et al. [7] were the pioneers who used the NRI to detect the role of preoperative TPN for malnourished patients undergoing surgical interventions. Similarly, the Veterans Administration Cooperative Group study of perioperative parenteral nutrition, successfully stratified operative morbidity and mortality using NRI [8]. NRI is a useful tool to evaluate patients at nutritional risk during hospital admission. It was found to be a sensitive, specific, and positive predictor for identifying patients at risk of developing complications after surgery [[9], [10], [11]]. Similarly, BMI also called Quetelet's index is also found to correlate with surgical outcomes [12]. Global analysis of BMI usage has shown an increase in trends in children and adolescents in a developing country [13]. Table 1a Compares BMI cutoffs for adults over 20 as per the WHO expert committee for the Asian population with our subjects [14]. Recently, several studies have reported that BMI has an impact on postoperative complications. But interestingly, obesity was not found to have prognostic implications for the long-term survival of the patients with gastric cancer [15,16]. The paradoxical ‘‘superior’’ outcomes in obese patients compared with normal patients are also described as ‘‘obesity paradox’’. This is in contrast to the commonly held belief that a high BMI is associated with an increased risk of death in the general population [17].

**Table 1a:** Comparison of our BMI distribution with BMI Cutoffs for Adults over 20 proposed by the WHO expert committee(15).

BMI Range kg/m2	Diagnosis	BMI Distribution
<16	Severe underweight	
16·0–16·9	Moderate	
17·0–18·49	Mild underweight	
<18.49	Underweight	<18.49 (n = 29)
18.5–24.99	Normal Range	18.5–24.99 (n = 96)
25–29.99	Overweight (pre-obese)	25–29.99 (n = 5)
≥30	Obese	>30 (n = 4)
30–39·9	Obese class I	
35–39·9	obese class II	
≥40	obese class III

**Table 1b:** Definition of postoperative complications.

Infectious	Non infectious
Wound infections: any redness or tenderness of the surgical wound with discharge of pus Abdominal abscess: Deep collection of Pus detected clinically and by Ultrasound/CT scan Pulmonary tract infection: Symptomatic with cough, fever, increased respiratory rate, decrease in oxygen saturation	Wound dehiscence: any dehiscence of fascia >3 cm Anastomotic leak: any dehiscence with clinical or radiological evidence of leak. Abdominal distension Number of days of stay Anastomotic leak vomiting

There is no agreement as to which index best reflects nutritional status in hospitalized patients. Various indicators like significant weight loss over time, low or high BMI, reduction in mid-arm circumference, and skinfold thickness have been used to determine nutritional risk. Bodyweight, for example, can be inaccurate if fluid balance derangements like edema or ascites are present, resulting in falsely high BMI measurements. Due to the inadequate performance of a single assessment tool, there is an increased tendency to combine diverse parameters. This increases the sensitivity and specificity to screen the high-risk malnourished patients [18]. In developing countries like Nepal, BMI is used primarily with age-independent cutoffs to identify chronic energy deficiencies or obesity in adults. According to one of the recent studies from Nepal, 17.27% of adults aged 18 years and above are underweight [19]. The practical implementation of nutrition-related guidelines is tedious and complicated. There is a need for a simple, achievable, cost-effective and objective nutritional assessment tool. Our study uses NRI and BMI as the nutritional screening tools in preoperative patients. The main objective of the study was to analyze NRI and BMI as a preoperative nutritional indicator in the determination of immediate postoperative outcomes in patients undergoing major gastrointestinal surgery. Similarly, to determine the correlation between 1) infectious and noninfectious complications and 2) benign and malignant diseases with NRI and BMI respectively.

## 2 Methods

It is an observational study carried out prospectively between July 2015 to May 2017 at the Department of Surgical Gastroenterology, NAMS, Bir Hospital, a tertiary level institution. This study was approved by the institutional ethical committee- “*IRB of NAMS, Bir Hospital*” and written consent was obtained from all of the patients. A comprehensive literature search published in English was done till 2019 using Hinari, PubMed, Cochrane Library, Web of Science, and ScienceDirect. This work has been reported in line with the STROCSS statement [20].

All the patients who underwent major GI surgery, under general anesthesia, were included in the study. Major was defined as the surgery involving >2hrs and excluded were an emergency major operation, gallstone disease operations, patients with cough, fever, and chest infections respectively. All the surgeries were carried out by the esteemed team of the department with an experience of more than 10yrs.

The sample size was calculated using the formula z^2^pq/d^2^ and reference of the calculation was taken from the study of R.D. Thieme et al. [21] where the detection rate of malnutrition by NRI was 88%. Twice the number of samples calculated was taken into consideration and the maximum tolerable error was taken as 5%.

The NRI is calculated using the formula: NRI = (15.9 × serum albumin g/L) + (41.7 × current weight/usual weight). The usual weight was defined as the stable weight 6 months before the illness. NRI >100 indicated that the patient is not malnourished, while 97.5–100 indicated mild malnourishment, 83.5–97.5 indicated moderate malnourishment and <83.5 indicated severe malnourishment respectively [7]. Similarly, BMI is defined as the weight in kilos divided by the square of height in meters [12].

BMI and NRI were two independent categorical variables. The dependent variables were the postoperative outcome in terms of infectious and noninfectious complications. All the preoperative parameters of the patients were recorded. The immediate postoperative complications were documented and categorized as infectious and noninfectious complications. The validated definition in accordance with “Clavien-Dindo classification system” was used as shown in Table 1b.

Data collection was done using the master chart. SPSS version 16 was used for the statistical analysis. Interim analysis was done after 6 months of the commencement of the research. The usefulness of the research was reviewed to determine whether the study should be terminated prematurely. In Multivariate analysis, ANOVA test was used to study the correlation between the dependent and the independent variables. A Chi-square test was used for categorical variables to analyze the dependent and the independent variables. Detection of malnutrition and its correlation with the postoperative outcomes was made with each of the assessment tools. *p* < 0.05 was considered as statistically significant.

## 3 Results

### 3.1 Characteristics of patients

**Fig. 1:**
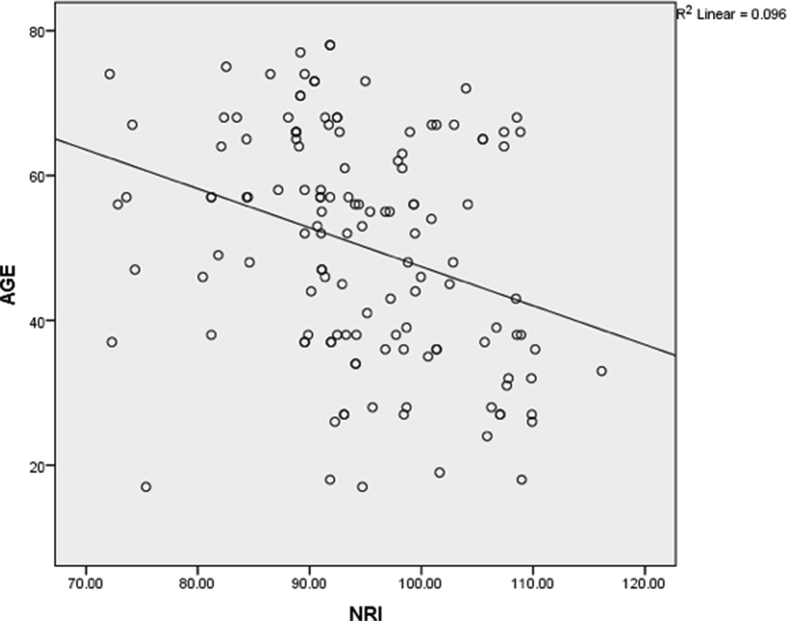
Scatter plot of linear correlation between NRI and Age. Table 2b and Fig. 1: Among 134 patients, the mean BMI was not statistically significant when correlated with the age of the patients. Whereas, NRI was found to have statistical significance (*p* < 0.05) with increasing age and showed significant negative correlation (correlation coefficient = −0.3100). The r^2^ = 0.096 indicates, with 10 fold increase in age the likelihood of malnutrition increases by 9.6%.

The total number of patients included in the study were 134. The mean age of the patient was 50.34 ± 15.891 and the maximum/minimum age of the patients was 78/17 years respectively. The mean BMI of the patient was 20.836 ± 3.537 where the mean BMI for the male patient was 19.67 ± 2.63 and for the female patient was 21.905 ± 3.92 respectively Table 2a. There was no statistically significant correlation between BMI and the age of the patients. And the correlation coefficient of −0.034 showed no statistical significance. Whereas, the mean NRI of the patient was 94.49 ± 9.164 where mean male/female patients were 93.32 ± 8.93/95.579 ± 9.305 respectively. There was a statistically significant correlation between NRI with the increasing age of the patients (*p* < 0.05). And the correlation coefficient of −0.3100 showed a significant negative correlation. The r^2^ of 0.096 showed that with the increase in the age there was a likelihood of malnutrition by 9.6% Fig. 1.(see Table 2b).

**Table 2a:** Distribution of BMI and NRI.

N = 134	Male (64)	Female(70)
Age	52.8 ± 15.012	48.14 ± 16.446
BMI	19.67 ± 2.63	21.905 ± 3.92
NRI	93.32 ± 8.93	95.579 ± 9.305

**Table 2b:** Correlation of age with BMI and NRI.

N = 134	Mean	Correlation coefficient	P value
BMI	20.836 ± 3.537	−0.034	0.693
NRI	94.49 ± 9.164	−0.3100	<0.05

The relationship between sex and complications was not statistically significant (*p* = 0.938). Also, the correlation coefficient of 0.007 showed no statistical significance. When correlating sex with BMI, the total numbers of males with abnormal BMI were 25 (39.06%) and the females were 13 (18.57%) respectively. And it was statistically significant (*p* = 0.009). Similarly, when correlating sex with NRI, 48 (75%) males and 44 (62.86%) females had abnormal values respectively. This distribution showed no statistical significance (*p* = 0.130) Table 3.

**Table 3:** Cross tabulation shows correlation of Sex with BMI and NRI.

	Sex	Normal	Abnormal	Total	*p* value
BMI	male	39(60.93%)	25(39.06)	64(100%)	0.009
	female	57(81.42%)	13(18.57%)	70(100%)	
	Total	96	38	134	
NRI	male	16(25%)	48(75%)	64(100%)	0.130
	female	26(37.14%)	44(62.86%)	70(100%)	
	Total	42	92	134	

Table 3: shows correlation of sex with BMI and NRI.

### 3.2 Postoperative complications

**Fig. 2:**
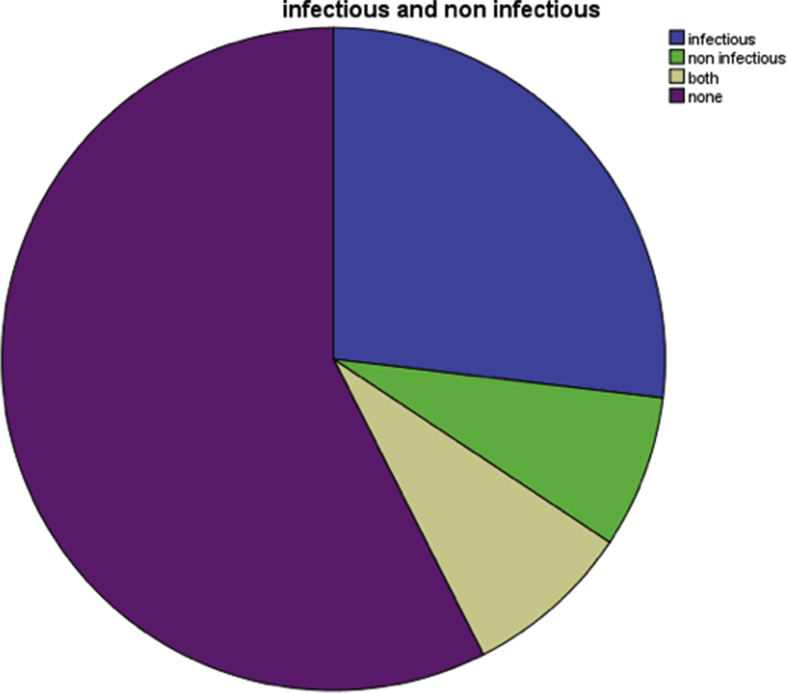
Distribution of infectious and non-infectious complications.

77 (57.46%) patients had both major and minor complications. Table 4 and Fig. 2 The infectious complications were not statistically significant across all the BMI subgroups (normal or abnormal, *p* > 0.05). Analysis of BMI and noninfectious complications showed no statistical significance. Subgroup analysis of NRI and complications showed statistical significance in severe malnutrition subgroup with NRI <83.5 (*p* < 0.006) Table 5.

**Table 4:** Overall complications.

Complications	N = 77 (57.46%)
Infectious	51 (66.23%)
Non infectious	26 (33.76%)
Total	77 (100%)

Table 4 and Fig. 2 shows out of 134 patients, 77 (57.46%) patients had complications after surgery. Out of which 51 (66.23%) patient had infectious complications and 26 (33.76%) had noninfectious complications.

**Table 5:** Complication and NRI subgroups.

NRI (n)	Complications (n)		*p* value
	No	Yes	
>100 (n = 37)	21 (56.75%)	16 (43.25%)	0.689
97.5–100 (n = 9)	3 (33.33%)	6 (66.66%)	0.170
83.5–97.5 (n = 75)	46 (61.33%)	29 (38.66%)	0.259
<83.5 (n = 13)	7 (53.84%)	6 (46.15%)	**0.006**
Total	77 (57.46%)	57 (42.53%)	

Table 5: The subgroup with severe malnutrition (NRI <83.5). showed statistical significance (p = 0.006) with the rate of complications. However, the rest of the NRI groups did not show any statistical significance. “n” = number.

### 3.3 Malnutrition and LOS

When evaluating BMI and the noninfectious complications, the group with the highest BMI showed a maximum number of LOS (22.5 ± 5.972 days). However, the mean number of LOS was not statistically significant across the groups (*p* = 0.078). When NRI and noninfectious complications were analyzed, the mean number of LOS was 17.615 ± 8.097. The maximum number of LOS in patients with severe malnutrition was not statistically significant. This could be due to the increased number of wound infections in the same subgroup of the patient that led to an increase in LOS.

### 3.4 Correlation between NRI and BMI and the prevalence of malnutrition

The maximum number of malignant cases fell in the subgroup where BMI was normal, 55 (57.29%) out of the total malignant case of 78 (58.20%). But the benign and malignant cases did not show any statistical significance with BMI. As with NRI, a total of 64 (47.76%) cases were diagnosed as malignant and fell in the abnormal NRI subgroup. And this group showed statistical significance (*p* = 0.004). Thus, the malignant disease was significantly associated with malnutrition according to NRI Table 6. The subgroup analysis showed the group with severe malnutrition had a significantly greater number of malignant cases (*p* = 0.003). The AUC of BMI and NRI was 0.515 and 0.463 respectively. Both were neither sensitive nor specific tests to identify the nutritional status correctly.

**Table 6:** Distribution of benign and malignant cases to normal and abnormal NRI.

	BMI		NRI	
	Normal	Abnormal	Normal	Abnormal
Benign	41(30.59%)	15(11.19%)	23 (17.16%)	33 (24.62%)
Malignant	55(41.04%)	23(17.16%)	14 (10.44%)	64 (47.76%)
*p* value	**0.072**	1	0.069	**0.004**

Table 6: In a subgroup of patients with malignant diseases, abnormal NRI was significantly associated with malnutrition (p = 0.004).

## 4 Discussion

Malnutrition in patients undergoing gastrointestinal (GI) surgery could be due to decreased oral food intake, chronic disease, tumor cachexia, impaired absorption in intestinal obstruction, and history of surgical bowel resection [22,23]. And these patients are at risk of increased morbidity and mortality, including a higher rate of infections, increased muscle loss, impaired wound healing, the longer LOS [[24], [25], [26]]. The goals of a nutritional assessment are to identify patients who are malnourished and to collect the information necessary to create a nutrition care plan as well as to monitor the adequacy of nutrition therapy [18].

We applied 2 different tools to a cohort of patients who underwent GI surgery. And we calculated the correlation between NRI and BMI with the incidence of postoperative morbidity. A high rate of patients was at a nutritional risk of 28.35% (38 out of 134) in our cohort as calculated by BMI Table 1a. Whereas, NRI detected 72.38% (97 out of 134) patients at nutritional risk Table 5. Similar results were seen in the study published by Pablo et al. [18] - 90% and Almeida AI et al. [27] - 87%. In our study, the number of malignancies in the abnormal group of the patients according to NRI was 64 (47.76%). Since the study was carried out in a developing country, there is a maximum chance of patients falling into the malnourished group 72.38% (97 out of 134).

The postoperative complications were 30.59% (41 out of 97) in nutritional risk group whereas in 11.94% (16 of 37) without nutritional risk Table 5. This result showed no statistical significance (*p* = 0.444). Similarly, no correlation between BMI and postoperative complications were seen. Whereas, the wound infection rate was statistically significant (*p* = 0.003) in severe malnutrition group as shown by NRI. In contrast, the LOS did not show any statistical significance with NRI (*p* = 0.782). However, the maximum number of LOS was 22.5 ± 5.97 days and was seen with the highest BMI score as compared to other groups although it was not statistically significant (*p* = 0.078). In surgical patients, Curtis LJ et al. [3] found moderately malnourished patients LOS were 18% longer on average than well-nourished patients and cost was 34% higher than for well-nourished patients with similar characteristics. Severely malnourished patients stayed 34% longer and had 38% higher total costs than well-nourished patients. Similarly, longer LOS 22.5 ± 5.97 days in >30 BMI in severely malnourished patients were seen but it was not statistically significant. The mean BMI in our study was 20.836 ± 3.537 and when it was correlated with age, the correlation coefficient was −0.034 with *p* = 0.693. Which showed no statistical significance. However, the mean NRI was 94.49 ± 9.164 and when it was correlated with the age, the correlation coefficient was - 0.31 with r^2^ of 0.096 and the *p* < 0.05 which was statistically significant.

When benign and malignant diseases were undertaken, the results of the present study suggested greater impairment of nutritional status in patients with malignant diseases assessed by the NRI, which was statistically significant, *p* = 0.04. Sciesser et al. [26], verified the higher prevalence of nutritional risk in patients with cancer as compared to benign illness. And similarly, Pablo et al. [18] demonstrated that patients with cancer had significantly higher weight loss. In our study, malnourished patients were significantly older and had longer LOS than well-nourished patients. In terms of benign and malignant diseases, there was no correlation between malnutrition and BMI (*p* = 0.072). However, there was statistical significance (*p* = 0.004) when NRI was used as the assessment tool Table 6.

In the current era of ERAS, nutritional screening, nutrition care-planning, and supplement of nutrition to the malnourished patients are emphasized to reduce the post-operative complications and LOS [28,29]. Recent, ASPEN guideline also recommends correction of macro as well as micronutrients after the surgery to reduce the post-operative complications [30]. Similarly, the latest RCT has indicated a significant role of pre-habilitation in high-risk patients [31,32]. It improves the cardiopulmonary reserve and thus better adapts the body for the physiological stress produced by the surgery [33].

It was found that home-based preoperative training programs effectively decreased the LOS and cost of treatment [34]. Not only preoperative but also post-operative and post-discharge nutritional supplements and counseling were found to have an impact on the outcome following surgery [35]. New technologies like computer software and mobile apps have been implemented to diagnose and monitor malnutrition [36]. Similarly, the result of a multicenter randomized controlled phase IV trial for the preoperative use of novel immune-nutrition in liver resection for cancer is awaited [37]. Even though, these innovations sound futuristic it is currently not feasible in the context of developing countries. We still depend on simple nutritional screening tools that are easily and readily available. With tools like NRI and BMI, we can easily segregate the high-risk patients and reduce the postoperative complications, LOS as well as a financial burden.

For a balanced discussion, we must include the NURIMAS Pancreas trial. This trial used 11 scores in surgical patient populations including NRI. Remarkably, the authors found no correlation between any of the scores with malnutrition and post-operative complications. So the study concluded that these scores may be discarded [38]. It could be due to prolonged retrospective recruitment that increased the effect of confounding factors. Patients at risk of malnutrition may have been monitored more closely and a larger number of postoperative complications identified, resulting in detection bias. And, pancreatic surgery itself has an inherent risk of complications and malnutrition could only be a secondary factor [39,40]. AUC of BMI and NRI (0.515 and 0.463) in our study also concluded both were neither sensitive nor specific tests to identify the nutritional status correctly. Our study may have a potential recruitment bias due to heterogeneous group and it may limit the generalizability. Similarly, several factors other than nutritional status like immune-physiological statuses and conditioning can also affect the outcome. Also, the NRI result might overestimate malnutrition, as albumin level might fluctuate with disease severity and the usual weight that is defined as the stable weight 6 months before the illness is informed by the patient which could be subject to bias. Similarly, BMI may give wrong values in fragile malnourished hospitalized patients with deranged fluid balance like edema or ascites. So, it is very important to have a sound clinical judgment along with the use of these tools and their significance.

## 5 Conclusion

The statistical analysis of NRI and BMI advocated: 1) NRI better correlates with postoperative wound infections and the LOS; 2) NRI showed that patients with advanced age and malignant diseases were at higher risk of malnutrition and postoperative complications. 3) And, AUC indicated both were neither sensitive nor specific tests. Patients under risk may benefit from preoperative nutritional support that helps to reduce postoperative morbidity, LOS, and cost. Further research is warranted that focuses on diagnostic tools for high-risk patients that can help in the management of malnourished surgical patients.

## Provenance and peer review

Not commissioned, externally peer reviewed.

## Authorship

Nabin Pokharel and Gaurav Katwal are the first authors and Subodh Kumar Adhikari is the co-author.

## Author disclosure

The authors have nothing to disclose.

## Ethical Approval

Ethical Approval had been taken from the IRB of National Academy of Medical Sciences, Bir Hospital and has been uploaded as a supplementary document.

## Sources of funding

The study had been supported by the institutional fund.

## Author contribution

The authors’ responsibilities were as follows: NP and GK are the first authors. NP designed, conducted the research and was responsible for data collection. NP and GK analyzed, interpreted the data and wrote the manuscript. SK-A is the co-authors, who critically revised the papers. And SK-A finally approved the manuscript.

## Registration of research studies

Name of the registry: ClinicalTrials.gov.

Unique Identifying number or registration ID: NCT04039035.

Hyperlink to the registration (must be publicly accessible): https://clinicaltrials.gov/ct2/show/NCT04039035?cntry=NP&rank=1.

## Guarantor

IRB of the National Academy of Medical Sciences, Bir Hospital, AND All the authors.

## Consent

It was a observational study and no intervention was done in the patient during the research. However, written and verbal consents had been taken from the patients/family who were included in the study. In case, respected Editorial board wants to see the consent, we can email all the consents that were taken.

## Declaration of competing interest

There are no conflict of interests in the submission of this manuscript, and the manuscript is approved by all the authors for publication. My institute “National Academy of Medical Sciences” representative is fully aware of this submission.
